# Three-Dimensional Whole-Organ Characterization of the Regional Alveolar Morphology in Normal Murine Lungs

**DOI:** 10.3389/fphys.2021.755468

**Published:** 2021-12-08

**Authors:** Mauricio A. Sarabia-Vallejos, Pedro Ayala-Jeria, Daniel E. Hurtado

**Affiliations:** ^1^Faculty of Engineering and Technology, Universidad San Sebastián, Santiago, Chile; ^2^Department of Respiratory Diseases, School of Medicine, Center of Medical Research, Pontificia Universidad Católica de Chile, Santiago, Chile; ^3^Department of Structural and Geotechnical Engineering, School of Engineering, Pontificia Universidad Católica de Chile, Santiago, Chile; ^4^Schools of Engineering, Medicine and Biological Sciences, Institute for Biological and Medical Engineering, Pontificia Universidad Católica de Chile, Santiago, Chile; ^5^Millennium Nucleus for Cardiovascular Magnetic Resonance, Santiago, Chile

**Keywords:** alveolar morphology, pulmonary porosity, alveolar surface density, surface-to-volume ratio, tissue dehydration methods

## Abstract

Alveolar architecture plays a fundamental role in the processes of ventilation and perfusion in the lung. Alterations in the alveolar surface area and alveolar cavity volume constitute the pathophysiological basis of chronic respiratory diseases such as pulmonary emphysema. Previous studies based on micro-computed tomography (micro-CT) of lung samples have allowed the geometrical study of acinar units. However, our current knowledge is based on the study of a few tissue samples in random locations of the lung that do not give an account of the spatial distributions of the alveolar architecture in the whole lung. In this work, we combine micro-CT imaging and computational geometry algorithms to study the regional distribution of key morphological parameters throughout the whole lung. To this end, 3D whole-lung images of Sprague–Dawley rats are acquired using high-resolution micro-CT imaging and analyzed to estimate porosity, alveolar surface density, and surface-to-volume ratio. We assess the effect of current gold-standard dehydration methods in the preparation of lung samples and propose a fixation protocol that includes the application of a methanol-PBS solution before dehydration. Our results show that regional porosity, alveolar surface density, and surface-to-volume ratio have a uniform distribution in normal lungs, which do not seem to be affected by gravitational effects. We further show that sample fixation based on ethanol baths for dehydration introduces shrinking and affects the acinar architecture in the subpleural regions. In contrast, preparations based on the proposed dehydration protocol effectively preserve the alveolar morphology.

## Introduction

Ventilation and perfusion are vital processes to facilitate gas exchange at the alveolar level, which is the primary function of the respiratory system. Pulmonary ventilation is defined as the process where air enters and leaves the alveolar units, which supplies with O_2_ to the alveolus and removes the expired CO_2_. Perfusion refers to the blood flow in the capillaries that surround the alveolar surface, which is fundamental for gas transport. The relationship between both processes is one of the cornerstones of respiratory physiology, as it not only allows us to understand the mechanisms underlying respiration but also explains the genesis and evolution of diseases such as hypoxemia and pulmonary emphysema, among others (Bajc and Jonson, 2011; Jögi et al., 2011).

Ventilation and perfusion have long been associated with the alveolar architecture, constituting another clear example of the celebrated structure-function paradigm in physiology. To maximize gas exchange between alveoli and capillaries, the mammalian lung takes on a highly porous structure that maximizes the perfused alveolar surface and, at the same time, maximizes the alveolar airspace volume (Hsia et al., 2016). Alterations in the balance between the alveolar surface and the alveolar airspace constitute the pathophysiological basis of chronic respiratory diseases such as pulmonary emphysema. In emphysematous lungs, the rupture of alveolar walls results in a marked decrease in the alveolar surface available for perfusion and gas exchange and in the loss of alveolar tissue recoil, ultimately deteriorating the respiratory function (Suga et al., 2010). This highlights the importance of characterizing the morphology of the alveolar tissue in the lung and elucidates how it influences lung function and pulmonary performance (Weibel, 2017).

From a morphological point of view, alveolar ventilation is associated with porosity, defined as the ratio between the volume of the alveolar cavity (airspace volume) divided by the nominal (reference) volume of lung tissue. Similarly, perfusion can be associated with alveolar surface density, defined as the ratio between the alveolar surface area over the nominal volume of lung tissue (Hsia et al., 2016). It is important to note that both definitions are independent of each other, as the alveolar cavity volume and surface are not necessarily related. Given its close relationship with the gas exchange process, the study of the spatial distribution of morphological parameters such as porosity and alveolar surface density provides a quantitative evaluation that can be related to ventilation and perfusion with regional resolution (Soldati et al., 2014; Clark et al., 2019).

To date, the characterization of alveolar morphology has been difficult due to its micrometric size and intricate architecture. Advances in micro-computed tomography (micro-CT) techniques have allowed the study of the shape and structure of pulmonary acini with high resolution and less destructively than traditional histological methods (Langheinrich et al., 2004; Vasilescu et al., 2012b). Besides, micro-CT has enabled the three-dimensional visualization of the acinar structure with high accuracy, which motivated a volumetric characterization of alveoli (Litzlbauer et al., 2006). Current morphometric studies of the lung tissue have analyzed the acinar morphology in terms of alveolar volume, alveolar diameter, surface-to-volume ratio, and porosity, among other parameters (Parameswaran et al., 2009; Vasilescu et al., 2012a; Concha et al., 2018; Sarabia-Vallejos et al., 2019). In particular, porosity and alveolar surface density emerge as insightful parameters in the study of diseases such as pulmonary emphysema (Yuan et al., 2010), as they quantify the evolution of abnormally large airspaces produced by alveolar enlargement. In effect, septum rupture in emphysema results in higher porosity and lower density of the surface area than those found in normal lungs, which directly affects the ventilation–perfusion ratio, making it challenging to exchange gases with the bloodstream (Parameswaran et al., 2009).

While morphometric studies reported in the literature have provided vital information about the structural parameters of the lung parenchyma, current knowledge is based on a small number of micrometric samples that are randomly located in the lung. Such localized information does not provide information on the spatial distribution of alveolar structural properties throughout the organ (Hsia et al., 2010). Based on this limitation, the scientific question that guides our work is: How is the regional distribution of morphological parameters in the whole lung? To answer this question, in this work, we combine micro-CT image acquisition, advanced image processing techniques, and computational geometry methods to unveil the three-dimensional spatial distribution of porosity, alveolar surface density, and surface-to-volume ratio in normal rat lungs. We also assess the effect of current gold-standard dehydration methods in the preparation of lung samples and their impact on related morphological parameters and propose a novel fixation protocol that considers the application of a methanol-PBS solution before hydration.

## Materials and Methods

The bioethics committee of the Pontificia Universidad Católica de Chile approved the following protocol. Nine adult Sprague–Dawley rats (∼300 g, sex-matched) were randomly assigned to three experimental groups according to the fixation method (see below, each group with *N* = 3). Subjects were kept under controlled humidity, light, and temperature conditions before the lung *in situ* fixation step. Food and water were provided *ad libitum* during this period.

### Lung Sample Preparation

The preparation of lung samples consisted of three subsequent steps: *in situ* fixation of the lung, *ex vivo* fixation of the lung, and dehydration of the lung sample. For the *in situ* fixation stage of the lung, we followed the protocol described by Hausmann (2007). Subjects were anesthetized with an intraperitoneal injection of ketamine and xylazine (30 mg^–1^ kg^–1^, Drag Pharma Invetec S.A., Santiago, Chile, and 5 mg^–1^ kg^–1^, Alfasan, Woerden, Holland, respectively). A cannula with a three-way in-line valve was introduced through the trachea of each subject in the supine position and was subsequently sealed using a cuff to instill into the lungs a formalin phosphate-buffered saline (F-PBS) solution at 4%. During the installation process, the pressure across the respiratory system was maintained at 20 cm H_2_O for 30 min using a syringe with a pressure transducer (AG Cuffil, Hospitech Respiration Ltd., Kfar Saba, Israel). Then, the three-way valve was closed to maintain pressure in the lungs, and the animal was refrigerated at 4°C for 8 h.

For the *ex vivo* lung fixation step, subjects were removed from the refrigerator, after which a median sternotomy was performed to remove the lungs out of the rib cage. During the whole surgery, care was exercised to avoid puncturing the organ, preventing leakage of the fixative solution. The left lung was dissected with the left bronchus clamped and then immersed into an F-PBS bath for 24 h. Only the left lung was further considered for analysis due to the sample size restrictions imposed by the micro-CT platform.

For the dehydration step, three different drying methods were assessed, which define the three experimental groups in this study. The drying methods were:

• Standard alcohol fixation (SAF): This method is the gold standard in histology and pathology (Hausmann, 2007; Braber et al., 2010). The sample was immersed for periods of 2 h in subsequent baths with increasing ethanol graduations (70, 80, and 90% ethanol in PBS), and finally in a 100% ethanol bath for 12 h. After this, the lung was removed from the last bath and left on a semi-covered plastic container to let it dry under ambient conditions for 3 h, to eliminate the remaining ethanol by evaporation.
• Modified alcohol fixation (MAF): Our research group designed this method as an alternative to the SAF method. First, the sample was immersed for 2 h in a 70% methanol-PBS solution, which is the main difference between SAF and MAF. Subsequently, the same steps described in the SAF fixation method were performed. It is important to remark that the action of methanol is different than ethanol. While ethanol only removes water from the tissue, methanol increases cellular permeability, thus allowing an enhanced alcohol diffusion during the posterior dehydration step.
• Standard alcohol fixation and HMDS (SAF-HMDS): This fixation method is recommended by the micro-CT manufacturer (Bruker-MicroCT, 2016). The sample was treated following the protocol in the SAF method. As a final and additional step, the sample was immersed in a hexamethyldisilazane solution (HMDS) for 2 h, after which the sample was allowed to dry under ambient conditions on a semi-covered plastic container for 3 h.

To characterize the volumetric change associated with the drying methods, the displaced volume of fluid was measured for each subject at the end of the dehydration stage and at the beginning of the *ex vivo* fixation stage. From these volumes, the lung volume ratio was calculated for each subject. Volume ratio values below 100% imply that the fixation and dehydration process resulted in sample shrinking.

### Micro-Computed Tomography Scanning Protocol and 3D Image Reconstruction

**FIGURE 1:**
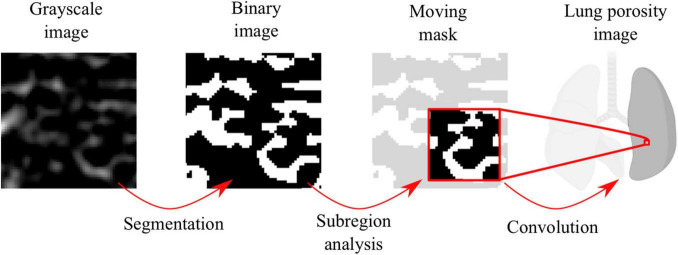
Workflow for the construction of alveolar porosity maps.

All lung samples obtained were scanned using a commercial micro-CT (SkyScan 1272, Bruker Inc., Kontich, Belgium). During imaging, the samples were placed on the sample plate with the axial axis of the lung vertically aligned. The voltage and current of the X-ray source were set at 10 kV and 250 μA, respectively. Pulmonary tomographic images were obtained using two voxel resolutions: isotropic 15 μm (low resolution) and isotropic 4 μm (high resolution). The first low-resolution acquisition was used as a scout scan to confirm that the sample fixation step did not introduce errors such as regions with marked alveolar collapse. The second high-resolution acquisition was used to generate images with an accurate definition of the alveolar architecture. Images were reconstructed using NRecon software (Bruker Inc., Kontich, Belgium) where misalignment compensation, ring artifact reduction, hardening, and Kuwahara filters were used to improve the signal-to-noise ratio. The acquired images were processed using median and Wiener filters to reduce the inherent noise, as well as a mix of top-hat and bottom-hat filters and histogram equalization to improve contrast, which delivered 3D grayscale images of the lung. For the morphological quantification of the images, grayscale images were segmented using a threshold filter on the Hounsfield unit scale based on the Otsu method (Xu et al., 2011) to obtain 3D binary images of the alveolar microstructure (see Figure 1). The intensity value in a voxel of the binary image was set equal to 1 if the voxel corresponded to tissue, or to 0 if the voxel corresponded to air.

Three-dimensional cubic domains of representative volume elements (RVE) with a border size of ∼500 μm were selected from the enhanced images of the lung. During the selection procedure, acinar regions were targeted, and zones with large portions of bronchi or blood vessels were avoided. For each experimental group, 9 RVEs were selected per subject (3 in each region of the lung; basal, mid, and apical), resulting in a population of 27 RVEs per group. RVE images were then segmented to obtain binary masks, which were the basis for the morphological analysis.

### Morphological Analysis, and Construction of 3D Porosity and Alveolar Surface Density Maps

The following morphological parameters were calculated for each RVE analyzed in this study: surface-to-volume ratio, mean alveolar diameter, alveolar wall thickness, porosity, and alveolar surface density. Parameter quantification was carried out using an in-house code written in Matlab (MathWorks, Version R2017a, Natick, MA, United States). The determination of the alveolar diameter was performed using the Sphere-fit method (Lesouple et al., 2021), which fits spheres within a point cloud using a least-squares algorithm. From the spheres obtained, an active contour algorithm was used to determine the surface and volume of the alveolar cavity (Strzelecki et al., 2013; Aganj et al., 2018). The ratio between these values allowed us to determine the surface-to-volume ratio for each alveolar cavity. The thickness of the alveolar wall was obtained by subtracting the alveolar radius of two contiguous spheres and the separation between the centers of these spheres. For each RVE, the global porosity was computed as the ratio between the volume of the alveolar cavities and the total RVE volume (reference volume).

Three-dimensional porosity maps were computed following the workflow sketched in Figure 1. Using binary images as a starting point, we constructed a moving 3D mask centered around each voxel of the lung image. The value of voxels inside the mask was set equal to 1, while voxels outside the mask were set equal to 0. For each lung image voxel, the associated mask was convoluted with the binary image to obtain the mask tissue volume, measured as the total number of non-zero voxels inside the mask. The mask airspace volume was computed as the difference between the total mask volume and the mask tissue volume. Finally, the porosity associated to one voxel in the lung image was determined as the ratio of mask airspace volume over the mask total volume. The final voxel porosity took values between 0 and 1, where 0 corresponded to a region only composed by airspace and 1 corresponded to a region only occupied by tissue with no gas. To assess the dependence on the choice of the mask size, we considered the results for five different mask sizes (140, 105, 70, 35, and 17.5 μm) when computing the porosity maps for the same segmented image. The resulting porosity maps were used to construct frequency histograms, which were then represented using kernel density estimation techniques to enable a direct comparison between all mask size cases.

**FIGURE 2:**
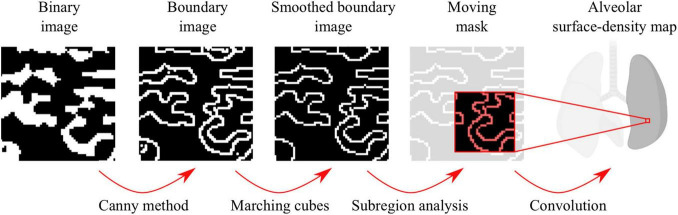
Workflow for the construction of alveolar surface-density maps.

Three-dimensional maps of alveolar surface-density maps were computed based on the workflow sketched in Figure 2. Using binary images as the starting point, the boundaries between alveolar tissue and airspace were detected using the Canny method for edge detection (Canny, 1986). To improve the boundary accuracy, the Marching Cubes algorithm (Zhao et al., 2018) was applied to obtain a smooth representation of the tissue-airspace boundary. To compute the surface area of the tissue-airspace boundary, we employed a level-set segmentation method (Vasilescu et al., 2012a; Magee et al., 2013) implemented in Matlab (Li et al., 2011). Finally, for each voxel in the lung image, the tissue-airspace surface area inside the moving mask around the voxel was obtained by convoluting the mask image with the smoothed boundary image, from which the surface density was obtained as the total surface area inside the mask divided by the volume of the mask. To assess the dependence of alveolar surface-density maps on the choice of the mask size, a sensitivity analysis similar to the one described for the case of porosity was carried out using the same mask size range.

Surface-to-volume ratio maps were constructed using the information from alveolar surface-density and porosity maps. For this purpose, let *A*_*alv*_, *V*_*airspace*_, and *V*_*ref*_ be the alveolar surface area, the alveolar airspace volume, and the reference volume of the cubic sample to be analyzed (RVE or moving mask), respectively. The surface-to-volume ratio (ρ) is then defined as the ratio of the alveolar surface area over the airspace enclosed by this surface, i.e.,


(1) $$\text{ρ}=\frac{A_{a\,l\,v}}{V_{a\,i\,r\,s\,p\,a\,c\,e}}.$$

Considering a reference cubic region whose volume is *V*_*ref*_, the alveolar surface area inside the reference volume can be estimated from the alveolar surface density (η) as


(2) $$A_{a\,l\,v}=\eta \cdot V_{r\,e\,f},$$

and the alveolar airspace volume for the same reference volume can be obtained from the porosity value (ϕ) as


(3) $$V_{a\,i\,r\,s\,p\,a\,c\,e}=\text{ϕ}\cdot V_{r\,e\,f}.$$

Substituting Eqs 2, 3 into the definition of surface-to-volume ratio described in (1), we obtain the relation


(4) $$\text{ρ}=\frac{\text{η}}{\text{ϕ}}.$$

Using Eq. 4, surface-to-volume ratio maps can be constructed from the porosity and the alveolar surface-density maps in a voxel-wise way. Eq. 4 can also be used to estimate the surface-to-volume ratio in the RVEs considered in the analysis.

To assess the regional distribution of alveolar porosity, alveolar surface density, and surface-to-volume ratio, regions of interest (ROI) were defined along the ventral-dorsal direction of each subject, following a method similar to that used in the regional characterization of lung deformation (Cruces et al., 2019; Hurtado et al., 2020). The regions of interest are connected sets of voxels selected from advancing planes in the selected direction, to achieve 10 contiguous regions with the same volume. The regional value of porosity and alveolar surface density is obtained as the average of the values contained in each ROI.

### Statistical Analysis

To detect significant differences in the morphological parameters between the study groups, nine RVE samples were selected per subject from randomly chosen sectors of the lung, which generates a total of 27 RVE samples per group. The comparison between groups was performed using the Mann–Whitney two-sided *U* test, considering a *p*-value of 0.05 corrected by the Bonferroni method to allow the comparison between multiple groups.

For the inter-group comparison of regional values of porosity and alveolar surface density, three sections were selected per anatomical region (apical, mid, or basal) in each subject, which gives a total of nine samples for each ROI per group. The comparison between the same ROI in different groups was carried out using the Mann-Whitney two-sided *U* test, considering a *p*-value of 0.05 corrected by the Bonferroni method to allow the comparison of multiple groups. The error bars in figures show the standard deviation. The variability of porosity and alveolar surface density between different anatomical sections in a single lung was assessed using the analysis of variance (ANOVA) test for each of the subjects in the experimental groups studied (SAF, MAF, and SAF-HMDS), after confirming normality of the samples considered using the D’Agostino and Pearson test.

## Results

Values for the surface-to-volume ratio, mean alveolar diameter, alveolar wall thickness, porosity, alveolar surface density, and lung volume ratio are reported in Table 1. The SAF group was significantly different than the MAF and SAF-HMDS groups for the surface-to-volume ratio, mean alveolar diameter, porosity, and lung volume ratio. Further, the mean alveolar diameter of the SAF-HMDS group resulted in significant differences when compared to the SAF and MAF groups. No significant differences were detected between the three groups for the case of the alveolar wall thickness and the alveolar surface density.

**TABLE 1:** Alveolar morphological parameters for the representative volume elements (RVE) samples.

Group	Surface-to-volume ratio (mm^–1^)	Mean alveolar diameter (μm)	Alveolar wall thickness (μm)	Porosity	Alveolar surface density (μm^–1^)	Lung volume ratio (%)
SAF (*n* = 27)	89.5 ± 10.9*^†^	29.48 ± 3.96*^†^	6.92 ± 0.86	0.51 ± 0.05*^†^	44.31 ± 1.91	56 ± 12*^†^
MAF (*n* = 27)	67.7 ± 8.8*	40.51 ± 4.82*^#^	7.09 ± 1.08	0.65 ± 0.05*	42.05 ± 0.72	87 ± 3*
SAF-HMDS (*n* = 27)	61.6 ± 5.5^†^	54.68 ± 5.18^#^^†^	7.02 ± 0.45	0.65 ± 0.03^†^	40.83 ± 1.53	92 ± 2^†^

*Statistical significance between SAF and MAF methods (p-value ≤ 0.05).

*^#^Statistical significance between MAF and SAF-HMDS methods (p-value ≤ 0.05).*

*^†^Statistical significance between SAF and SAF-HMDS methods (p-value ≤ 0.05).*

**FIGURE 3:**
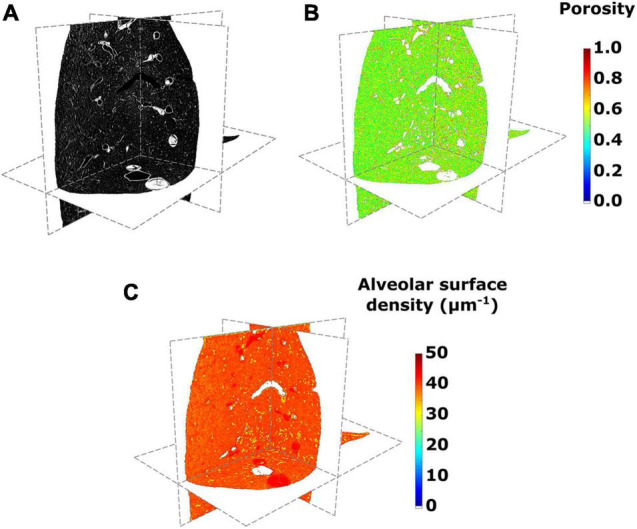
**(A)** Micro-computed tomography (CT) images of a lung in its axial, sagittal, and coronal views, **(B)** porosity, and **(C)** alveolar surface density maps for the SAF-HMDS group in the different anatomical planes.

Figure 3 shows the results from 3D micro-CT imaging processing and the spatial morphological analysis for the whole lung of a representative subject in the SAF-HMDS group. Micro-CT images of the whole lung displayed major airway and vasculature structures at the macroscopic level, as well as delivered detailed information of bronchioli, respiratory ducts, and acinar structures (see Figure 3A). The spatial distribution of porosity and alveolar surface density were visually found to be homogeneous throughout the entire domain of the lung (see Figures 3B,C, respectively).

**FIGURE 4:**
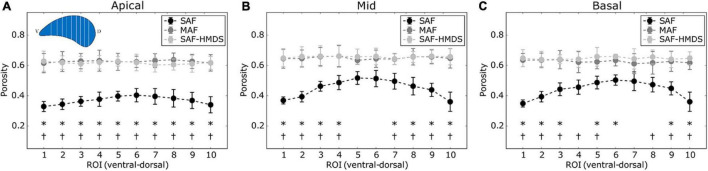
Regional distribution of porosity in the ventral-dorsal direction for three regions of the lung: **(A)** apical zone, **(B)** mid zone, and **(C)** basal zone. Significant differences (*p*-value ≤ 0.05) between the SAF and MAF, SAF and SAF-HMDS, MAF, and SAF-HMDS groups are indicated by *, †, respectively. Each group ROI considered *n* = 9 samples.

The regional distribution of alveolar porosity for the apical, mid, and basal zones of the lung is shown in Figures 4A–C, respectively. For the three areas analyzed, we found that the regional porosity values of the SAF group are significantly different (typically lower) than the values of the MAF and SAF-HMDS groups, with some exceptions in the mid and basal zones. No significant differences were found between the MAF and SAF-HMDS groups. Regarding the spatial distribution of porosity, uniform values were observed along the ventral-dorsal direction in all the areas analyzed in the MAF and SAF-HMDS groups. In contrast, a concave distribution is observed in the SAF group, with a tendency to reduce porosity toward the most ventral and dorsal areas.

**FIGURE 5:**
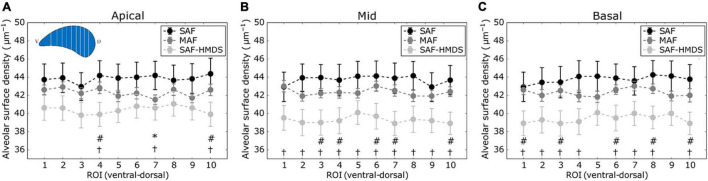
Regional distribution of alveolar surface density in the ventral-dorsal direction for three regions of the lung: **(A)** apical zone, **(B)** mid zone, and **(C)** basal zone. Significant differences (*p*-value ≤ 0.05) between the SAF and MAF, SAF and SAF-HMDS, and MAF and SAF-HMDS groups are indicated by *, †, and #, respectively. Each ROI datapoint represents the mean ± standard deviation of *n* = 9 samples.

The regional distribution of the alveolar surface density for the apical, mid, and basal areas of the lung is shown in Figures 5A–C, respectively. For the mid and basal cases, significant differences were observed between the SAF and SAF-HMDS groups for almost all ROIs. Furthermore, significant differences between the MAF and SAF-HMDS groups are observed for half of the ROIs in the same areas. In the three groups, a uniform distribution of values is observed along the ventral–dorsal direction, for the apical, mid, and basal zones.

**FIGURE 6:**
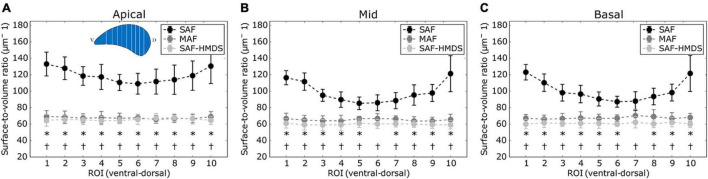
Regional distribution of surface-to-volume ratio in the ventral-dorsal direction for three regions of the lung: **(A)** apical zone, **(B)** mid zone, and **(C)** basal zone. Significant differences (*p*-value ≤ 0.05) between the SAF and MAF, SAF and SAF-HMDS, and MAF and SAF-HMDS groups are indicated by *, †, respectively. Each ROI datapoint represents the mean ± standard deviation of *n* = 9 samples.

The regional distribution of the surface-to-volume ratio for the apical, mid, and basal lung zones is shown in Figures 6A–C, respectively. With the particular exception of two ROIs, virtually all the regional values of the SAF group were found to be significantly different (higher) than the values of the MAF and SAF-HMDS groups. No significant differences were found between the MAF and SAF-HMDS. Furthermore, a uniform distribution of values is observed along the ventral–dorsal direction in all the areas analyzed for MAF and SAF-HMDS groups. In contrast, a convex distribution is observed in the SAF group, with a tendency to increase the surface-to-volume ratio values toward the most ventral and dorsal areas of the lung.

**FIGURE 7:**
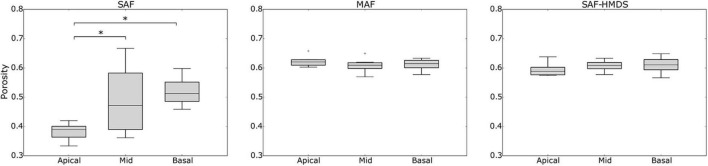
Single-lung (Subject 1) analysis of variability of alveolar porosity in different anatomical sections in Subject 1. Nomenclature: *p ≤ 0.05.

**FIGURE 8:**
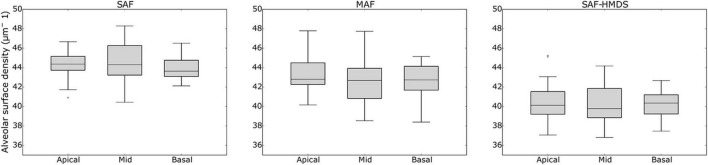
Single-lung analysis of variability of alveolar surface density in different anatomical sections in Subject 1. Nomenclature: *p ≤ 0.05.

When comparing the alveolar porosity in different sections of a single lung, no significant differences were found between the apical, mid, and basal sections for lungs in the MAF group (see Figure 7 for a graphical account of the results in Subject 1 and Supplementary Table 1 for the ANOVA results). In contrast, significant differences in alveolar porosity between anatomical sections were found in all of the lungs in the SAF group. For the case of alveolar surface density, no significant differences between sections were detected in lungs of the MAF and SAF-HDMS groups (see Figure 8 for Subject 1 and Supplementary Table 2 for the ANOVA results).

**FIGURE 9:**
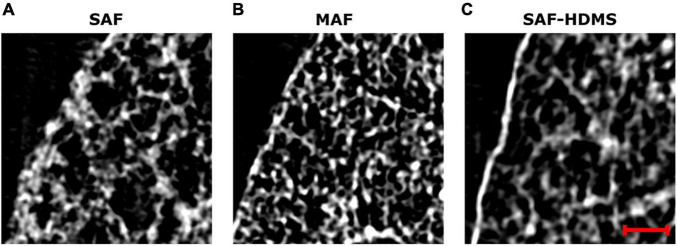
Magnifications of grayscale micro-CT images for subpleural regions of three representative subjects: **(A)** SAF group, **(B)** MAF group, and **(C)** SAF-HMDS group. The red scale bar corresponds to 100 μm.

The effects of mask size on the generation of porosity and alveolar surface density maps are reported in Supplementary Figures 1, 2. For both, porosity, and alveolar surface density, we observe that mask sizes above 70 μm result in unimodal histograms with similar characteristics (see Supplementary Figures 1F, 2F). In contrast, mask sizes smaller than 70 μm result in density functions that are not consistent with larger size masks, and that show oscillations in the range of smaller values. Figure 9 shows magnifications of a pleural sector for three representative subjects from each group, where pleural thickening is observed for the case representing the SAF group. In contrast, a thinner pleural thickness is observed in the MAF and SAF-HDMS group representatives.

## Discussion

In this work, we have studied the alveolar architecture of rat lungs using micro-CT and advanced computational geometry techniques. To the best of our knowledge, this work constitutes one of the first attempts to characterize the three-dimensional morphological parameters such as surface-to-volume ratio, porosity, and alveolar surface density in the whole lung of Sprague–Dawley subjects. One of the major findings is that regional porosity, alveolar surface density, and surface-to-volume ratio have a uniform distribution in normal lungs, which do not seem to be affected by gravitational effects.

Structural studies based on micro-CT imaging have focused on C57BL/6 murine lungs, both under normal and diseased conditions (Parameswaran et al., 2009; Vasilescu et al., 2012a), where the fixation procedure of the lung samples was similar to that performed in the SAF group. In 10-week-old normal mice, the mean alveolar diameter reported is 59 ± 2 μm (Parameswaran et al., 2009), which is comparable to the mean alveolar diameter found in the SAF-HMDS group in our work, and in the order of magnitude of the other two groups. Previous studies have shown that the alveolar volume and diameter in mice are smaller than in rats (Faffe et al., 2002). It is worth remarking that studies in mice use a higher tracheal pressure (30 cm H_2_O) during *in situ* fixation than the pressure considered in this work (20 cm H_2_O). More substantial tracheal pressures result in a larger alveolar expansion in mice, which may explain the similarity with the alveolar diameter values found in this work. The morphological analysis performed by Vasilescu et al. (2012a) in the same species delivered surface-to-volume ratio values of 52 ± 3.7 and 47.7 ± 6 mm^–1^ in young (12-week-old) and adult (91-week-old) subjects, respectively. These values are smaller than the ones found in this study for all groups (Table 1) but coincide in the order of magnitude. Similarly, Xiao et al. (2016) studied the acini of A/J mouse *in situ* using synchrotron-based micro-CT. They found surface-to-volume ratio values of 79.8 ± 8.9 mm^–1^, which are very close to those reported in this work in Table 1. We note that if the alveolus cavity is idealized as a sphere, then the surface-to-volume ratio is inversely proportional to the alveolar diameter. This, in turn, would imply that larger values of surface-to-volume ratio are to be expected in mice than in rats, which is not the case. Again, we attribute these differences to the high tracheal pressure applied in mice experiments (30 cm H_2_O), which results in larger alveoli dimensions. We note that in our work, the choice of applying an applied tracheal pressure of 20 cm H_2_O was made to target physiological values of tidal volume, as a tracheal pressure of 30 cm H_2_O typically corresponds to total lung capacity in murine subjects (Namati et al., 2006). The group of Litzlbauer et al. (2006) measures the alveolar surface density from micro-CT images using stereological methods for morphological quantification. In their study, left porcine lungs were fixed by using ventilation of formaldehyde vapors at 35 cm H_2_O. The alveolar surface density was measured as the alveolar surface area divided by the volume of interest, giving values close to the obtained in this study (between 30.5 and 35.5 mm^–1^).

Three-dimensional maps displaying the distribution of porosity in the lung were successfully constructed for all subjects (see Figure 3B for a representative case). The resulting maps showed a convergent distribution for mask sizes greater than 70 μm (see Supplementary Figure 1). Porosity in the pulmonary parenchyma was found to be regionally uniform everywhere in the lung and locally similar in the MAF and SAF-HMDS groups (see Table 1 and Figure 4). These results suggest that the spatial distribution of regional porosity is homogeneous and is not subject to gravitational effects. This conclusion is supported by previous studies, like the one reported by Hoffman et al. (Mullan et al., 1997; Namati et al., 2006). They estimated the air content on primate lungs in different anatomical regions, observing that air content is uniformly distributed in the lung and does not depend on the location of measurement. Another study that supports this conclusion is the work of Hogg et al., where 16 parenchyma samples were randomly dissected from different regions of frozen human lungs and analyzed using micro-CT (McDonough et al., 2015). The alveolar density, defined as the number of alveoli in a reference volume, was found to be uniform regardless of their original location in the lung.

In our study, a marked reduction in alveolar porosity was observed toward the subpleural regions in the SAF group (most dorsal and ventral zones, see Figure 4). Further, in every single lung of the SAF group, the porosity was found to be significantly different depending on the lung region (see Figure 7 and Supplementary Table 1). A careful examination of micro-CT images in those regions for a representative subject of the SAF-group revealed thickened alveolar septums, which is suggestive of alveolar collapse (micro-atelectasis) (see Figure 9A). In contrast, alveolar structures close to the pleura in representative subjects of the MAF and SAF-HMDS groups do not display alterations when compared to proximal acinar structures (see Figures 9B,C, respectively). These observations, along with the strong volume reductions observed in the SAF group (Table 1, lung volume ratio), suggest that the decrease in regional porosity in the SAF group is likely to be an artifact of the method rather than a physiological condition.

Similarly to the case of porosity, three-dimensional maps of alveolar surface density were obtained for all lung samples (see Figure 3C for a representative case). The resulting maps showed a convergent distribution for mask sizes greater than 70 μm (see Supplementary Figure 2). All three groups suggest that the distribution of alveolar surface density is homogeneous throughout the lung (see Figures 5, 8). Groups typically do differ in their assessment of ROI values. For example, significant differences between the SAF and SAF-HMDS groups were found in 21 out of 30 ROIs considered. More conclusive results were found in the study of the regional surface-to-volume ratio, where uniform distributions with similar values were found in the MAF and SAF-HMDS groups. These results suggest that the surface-to-volume ratio is homogeneous throughout the lung and does not exhibit a gravitational dependence. In contrast, the SAF group resulted in heterogenous distribution that largely deviated from the values found in the MAF and SAF-HMDS groups. We note that, since the surface-to-volume ratio is inversely proportional to the porosity, we conclude that the seemingly increasing values toward the most ventral and dorsal regions can be regarded as artifacts in the alveolar architecture induced the SAF method, based on the conclusions reached in the study of regional porosity.

Throughout this work, three methods for dehydration have been considered in the fixation of lung samples. The gold-standard and most popular method in studies involving the histological analysis and micro-CT imaging of murine lungs has been the SAF method, which employs ethanol solutions for the dehydration step (Puchtler et al., 1970; Hausmann, 2007; Braber et al., 2010). However, in our work, we have shown that the SAF method results in considerable lung shrinking (Table 1) that markedly affects the alveolar architecture in subpleural regions (Figure 9A). An alternative method is the SAF-HMDS, which is predominantly used to prepare samples for electron microscopy, with some applications in micro-CT sample preparations. One advantage of the SAF-HMDS method is that it allows a rapid drying that has been shown to preserve the alveolar morphology without significant alterations (Bray et al., 1993; Lee and Chow, 2012). However, the SAF-HMDS method has important operational disadvantages and risks to the user, as inhalation or skin exposure to HMDS is known to be hazardous to health (Chou and Chang, 2007). Another disadvantage is the management of HMDS residuals, as degradation results in products that can be harmful to the environment and that require special disposal procedures (Alleni et al., 1997). Here, we propose and assess the use of the MAF method as an alternative in the dehydration of lung samples. The MAF dehydration method has been commonly employed in molecular biology to quantify the presence of biomarkers and to detect specific genetic alterations in organs/tissues (Noguchi et al., 1997; Anami et al., 2000). However, to the best of our knowledge, its application in the preparation of samples for micro-CT analysis is novel and has not been reported in the literature. Our results show that the application of a methanol-PBS solution before subsequent baths of ethanol solutions in lung samples preserves their volume (Table 1) and alveolar architecture everywhere in the lung, as most of the morphological parameters analyzed in this work do not display substantial differences between the MAF and SAF-HMDS groups. We believe that the success of the MAF method is related to the ability of the methanol bath to increase cellular permeability, which then allows for enhanced diffusion properties during the ethanol bath dehydration step (Puchtler et al., 1970). We further note that methanol and PBS are safer in health terms than HMDS [lethal dose (LD_50_) and lethal concentration (LC_50_) values are considerably smaller for HMDS than methanol], and their disposal can be done without special requirements (the biodegradability of methanol is 99% while for HMDS is just 15.3%) (Sullivan and Cummins, 2005; Jang et al., 2019). Thus, we conclude that the proposed MAF dehydration method represents a convenient, sustainable, and safe procedure that does not alter the alveolar morphology in treated lung samples.

Several aspects of this work can be improved in future contributions. We have shown in our study that all fixation methods lead to different levels of tissue shrinking, which directly affects the alveolar architecture and the associated morphological parameters. Recently, *in vivo* micro-CT imaging of murine lungs has been reported (Lovric et al., 2017), where the acinar structure was reconstructed with high precision in living subjects. Future efforts on the morphological characterization of the lung may benefit from these *in vivo* imaging techniques, which may confirm or correct the values reported in this work. Another limitation of our study was the use of a single airway pressure level. Due to the elastic nature of the alveolar wall, the morphological values described in this work are expected to change in the event of different levels of airway pressure. Besides, our results have been obtained using only three subjects per group. While this small sample has allowed us to detect significant differences between groups, larger populations of Sprague–Dawley rats and other species should be considered in future works to confirm and extend our conclusions. Finally, in this work, we have advocated for the characterization of alveolar porosity, which is a microstructural parameter that is not commonly reported in respiratory physiology. We note, however, that porosity plays a crucial role in describing the mechanical response of porous biomaterials (Currey, 1988). A recent theoretical study shows that porosity, along with the alveolar wall elasticity, is the most relevant microstructural parameter in the mechanical response of the lung parenchyma (Concha et al., 2018; Concha and Hurtado, 2020). Further, the study shows that an increase in porosity, which can be directly associated with alveolar airspace enlargement, may signify a loss of parenchymal elastance, a mechanical relationship that has long been observed in lungs with pulmonary emphysema (Nagai et al., 1991). Therefore, a deep understanding of the porosity distribution in the whole lung plays a vital role in the creation of microstructurally-informed constitutive models (Eskandari et al., 2019; Álvarez-Barrientos et al., 2021) that can predict the overall properties of the lung, as well as in informing organ-level computational models of the respiratory system (Eskandari et al., 2015).

## Data Availability Statement

The raw data supporting the conclusion of this article will be made available by the authors, without undue reservation.

## Ethics Statement

The animal study was reviewed and approved by the Bioethics Committee of the Pontificia Universidad Católica de Chile.

## Author Contributions

MS-V and PA-J carried out the experiments. MS-V and DH designed the computational methods, interpreted the results, and wrote the manuscript. MS-V wrote computer codes and performed image analysis and statistical analysis. MS-V, PA-J, and DH designed the experiments, reviewed the final manuscript, contributed to the article, and approved the submitted version.

## Conflict of Interest

The authors declare that the research was conducted in the absence of any commercial or financial relationships that could be construed as a potential conflict of interest.

## Publisher’s Note

All claims expressed in this article are solely those of the authors and do not necessarily represent those of their affiliated organizations, or those of the publisher, the editors and the reviewers. Any product that may be evaluated in this article, or claim that may be made by its manufacturer, is not guaranteed or endorsed by the publisher.
