# Supplementary material for: Three-Dimensional Whole-Organ Characterization of the Regional Alveolar Morphology in Normal Murine Lungs
Source: Front Physiol. 2021 Dec 8;12:755468. doi: 10.3389/fphys.2021.755468 (PMC8692792; doi:10.3389/fphys.2021.755468)
Supplement: Supplementary file 1 [file Data_Sheet_1.PDF]

Supplementary Material

Three-dimensional whole-organ characterization of the regional alveolar morphology in normal murine lungs

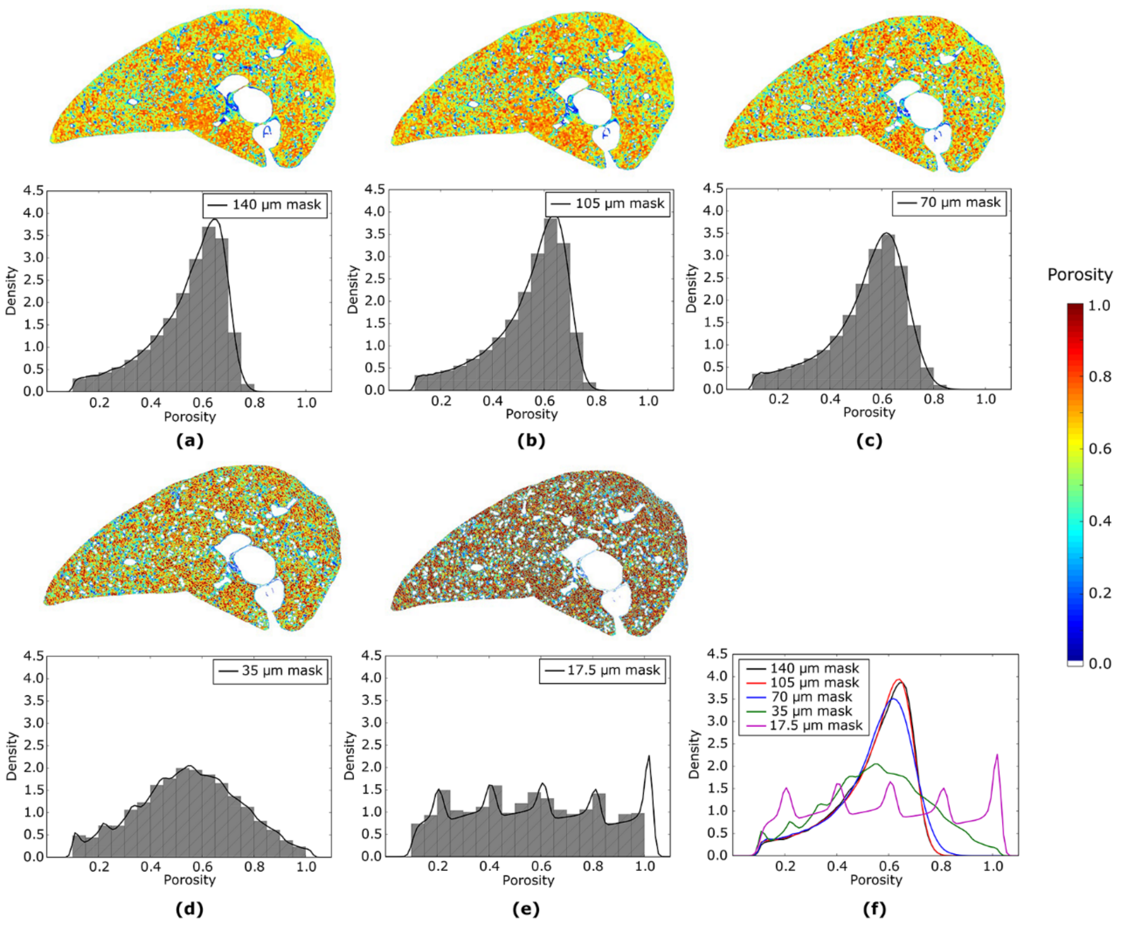

Figure 1s. Sensitivity analysis of the mask size in the generation of porosity maps. Histograms for the medium zone of a representative subject from the MAF group were constructed from porosity maps that employed a mask size of **(a)** 140 µm, **(b)** 105 µm, **(c)** 70 µm, **(d)** 35 µm, and **(e)** 17.5 µm. Density functions fitted to histograms for all cases are shown in **(f)** for comparison purposes.

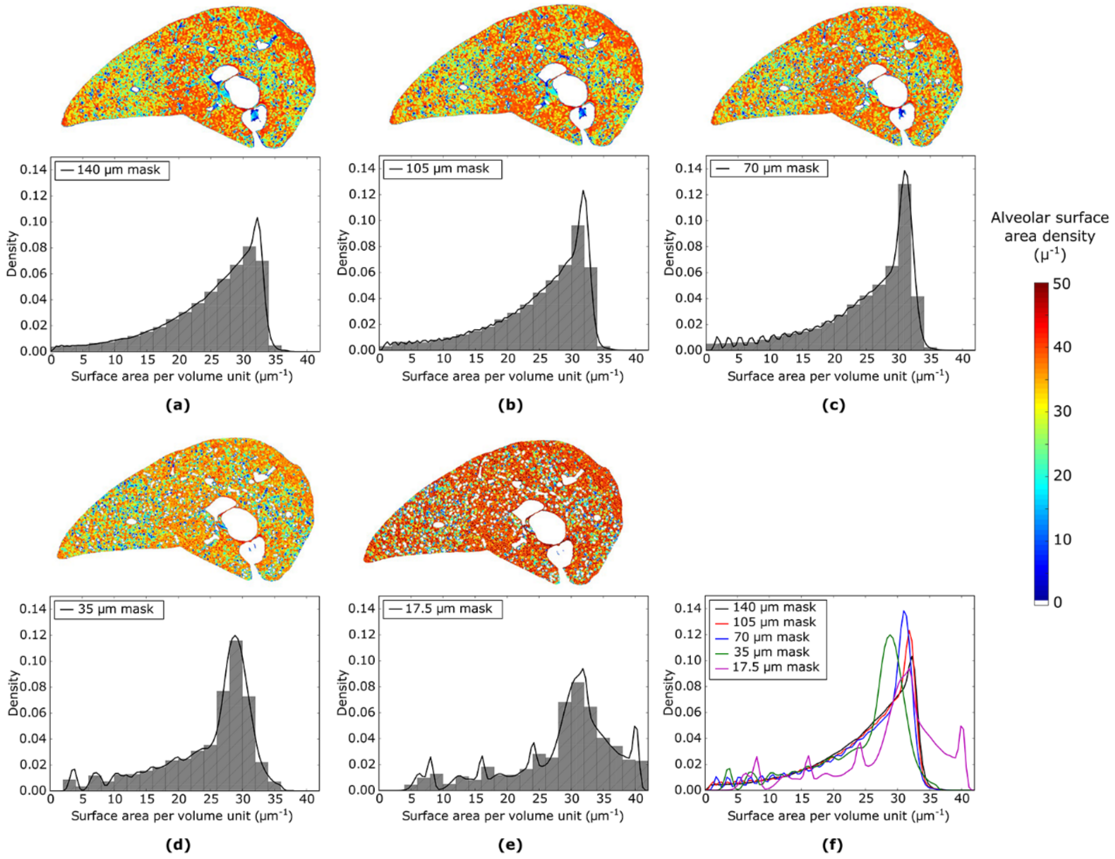

Figure 2s. Sensitivity analysis of the mask size in the generation of alveolar surface area density maps. Histograms for the medium zone of a representative subject from the MAF group were constructed from alveolar surface area density maps that employed a mask size of **(a)** 140 µm, **(b)** 105 µm, **(c)** 70 µm, **(d)** 35 µm, and **(e)** 17.5 µm. Density functions fitted to histograms for all cases are shown in **(f)** for comparison purposes.

Table 1s. ANOVA results for the analysis of lung porosity in single lungs of groups SAF, MAF, SAF-HMDS.

		ANOVA Summary					
		**Source**	**Degrees of Freedom (DF)**	**Sum of Squares (SS)**	**Mean Square (MS)**	**F-Stat**	**P-Value**
**SAF Group**	**L01**	Between Groups	2	0.0643	0.0321	49.605	0.0222
		Within Groups	15	0.0971	0.0065		
		Total:	17	0.1614			
	**L02**	Between Groups	2	0.0819	0.041	30.5011	0
		Within Groups	15	0.0201	0.0013		
		Total:	17	0.1021			
	**L03**	Between Groups	2	0.0718	0.0359	26.818	0
		Within Groups	15	0.0201	0.0013		
		Total:	17	0.0919			
**MAF Group**	**L01**	Between Groups	2	0.0007	0.0003	0.6786	0.5223
		Within Groups	15	0.0076	0.0005		
		Total:	17	0.0083			
	**L02**	Between Groups	2	0.0017	0.0009	1.1193	0.3523
		Within Groups	15	0.0117	0.0008		
		Total:	17	0.0134			
	**L03**	Between Groups	2	0.0014	0.0007	1.8254	0.1952
		Within Groups	15	0.0059	0.0004		
		Total:	17	0.0073			
**SAF-HDMS Group**	**L01**	Between Groups	2	0.0008	0.0004	0.6225	0.5499
		Within Groups	15	0.0093	0.0006		
		Total:	17	0.0101			
	**L02**	Between Groups	2	0.0028	0.0014	5.69	0.0145
		Within Groups	15	0.0037	0.0002		
		Total:	17	0.0065			
	**L03**	Between Groups	2	0.0003	0.0001	0.1214	0.8865
		Within Groups	15	0.0177	0.0012		
		Total:	17	0.018			

Table 2s. ANOVA results for the analysis of alveolar surface density in single lungs of groups SAF, MAF, SAF-HMDS.

		Source	Degrees of Freedom (DF)	Sum of Squares (SS)	Mean Square (MS)	F-Stat	P-Value
			**ANOVA Summary**				
**SAF Group**	**L01**	Between Groups	2	0.772	0.386	0.0679	0,9347
		Within Groups	15	85.3204	5.688		
		Total:	17	86.0924			
	**L02**	Between Groups	2	15.1533	7.5767	62.657	0.0105
		Within Groups	15	18.1383	1.2092		
		Total:	17	33.2917			
	**L03**	Between Groups	2	0.4137	0.2069	0.1187	0.8889
		Within Groups	15	26.1509	1.7434		
		Total:	17	26.5646			
**MAF Group**	**L01**	Between Groups	2	20.888	1.0444	0.2362	0.7925
		Within Groups	15	66.322	4.4215		
		Total:	17	68.4108			
	**L02**	Between Groups	2	60.506	3.0253	0.5072	0.6122
		Within Groups	15	89.4727	5.9648		
		Total:	17	95.5233			
	**L03**	Between Groups	2	60.104	3.0052	0.8977	0.4283
		Within Groups	15	50.2137	3.3476		
		Total:	17	56.2242			
**SAF-HDMS Group**	**L01**	Between Groups	2	0.9522	0.4761	0.1009	0.9047
		Within Groups	15	70.8119	4.7208		
		Total:	17	71.7641			
	**L02**	Between Groups	2	2.7754	1.3877	0.6373	0.5424
		Within Groups	15	32.6616	2.1774		
		Total:	17	35.437			
	**L03**	Between Groups	2	14.6665	7.3333	0.9183	0.4205
		Within Groups	15	119.7805	7.9854		
		Total:	17	13.4447			
